# Mr. Killer, on the Use of Digitalis in Hernia

**Published:** 1801-10

**Authors:** R. W. Killer

**Affiliations:** Manchester


### *To the Editors of the Medical and Phyfical Journal.*

Gentlemen,

It is to be lamented that the Reporters of medical cafes have fallen frequently into the error of afcribing to the operation of a fingle remedy what is due to the effect of feveral. Surely, we ought not to allow fterling merit to any particular remedy, till we have had fufficient experience of its efficacy in the removal of difeafe, unaided by any other eftablifhed mode of cure.

Into this error I conceive Mr. Simmons has fallen, in attributing the recovery of the patient with ftrangulated hernia, (whofe cafe was reported in the laft number but one of your excellent Journal,) to the exhibition of Digitalis. This cafe occurring in the Infirmary, I had an opportunity of attending to it, as one of the furgeons to that charity; and as my opinion militates againft the inference drawn by my colleague, refpecting the utility of the above medicine in the prefent inftance; and confidering that the very warm panegyric which is beftowed on this remedy may induce others to rely too much upon it in fimilar cafes, I take the liberty of offering the reafons for my diffent.

The patient was admitted under the care of Dr. Taylor, who directed feveral leeches to be applied to the fcrotum, a large dofe of calomel, and that he fhould go into the warm bath. By thefe means the volume of the tumour was confiderably diminifhed; however, as reduction was ftill impracticable, a confultation was held, and the plan of treatment, defcribed by Mr. Simmons agreed upon. A grain of Digitalis was given, and in a quarter of an hour after, and before it had produced any fenfible effect, a ftrong tobacco glyfter was thrown up. This brought on ficknefs and violent retchings inftantaneoufly, which continued till the patient was reduced to a ftate of afphyxia. Whilft in this condition the hernia was reduced with tolerable facility.

I fhould

I ſhould imagine that any perſon who has been in the habit of adminiſtering the Digitalis, muſt be convinced that *a single grain of it* has rarely, if ever, produced ſuch violent effects as took place in this inſtance. I cannot learn that ſuch an event ever happened in this infirmary, where it has long been preſcribed with great freedom. I will not deny that the addition of a ſedative power, like the Digitalis, might increaſe the conjoined debilitating effects of bleeding, the warm bath, and tobacco glyſters. But, I believe, few practitioners who have experienced the ſalutary influence of the latter remedies, will conſider the exhibition of the former as any other than a ſlender auxilliary to ſuch powerful means.

Mr. Simmons expects that this remedy will be a mean of cauſing the operation for incarcerated hernia to be leſs common on the continent, where, he obſerves, the ſurgeons perform it much oftener than in England. I moſt ſincerely wiſh his expectation may be realized; I am not a friend to haſty operations, yet it is generally admitted, that in conſequence of the continental ſurgeons having recourſe to the operation before too violent inflammation has come on, they ſucceed better in general than my own countrymen. My own experience and repeated obſervation, would lead me to infer that more patients are loſt in this complaint, by delaying the operation too long, than by its premature performance.

Should theſe obſervations be deemed worthy a page in your Journal, you will oblige by their inſertion,

*Manchester,* Your's, &c.

